# An Evaluation of Quantitative PCR Assays (TaqMan^®^ and SYBR Green) for the Detection of *Babesia bigemina* and *Babesia bovis*, and a Novel Fluorescent-ITS1-PCR Capillary Electrophoresis Method for Genotyping *B. bovis* Isolates

**DOI:** 10.3390/vetsci3030023

**Published:** 2016-09-13

**Authors:** Bing Zhang, Jacqueline L. Sambono, Jess A. T. Morgan, Bronwyn Venus, Peter Rolls, Ala E. Lew-Tabor

**Affiliations:** 1 Department of Agriculture & Fisheries, Agri-Science Queensland, Animal Science, Dutton Park QLD 4102, Australia; Bing.Zhang@daf.qld.gov.au (B.Z.); Jessica.Morgan@daf.qld.gov.au (J.A.T.M.); 2 Department of Agriculture & Fisheries, Biosecurity Queensland, Tick Fever Centre, Wacol QLD 4076, Australia; Jacqueline.Sambono@daf.qld.gov.au; 3 Queensland Alliance for Agriculture & Food Innovation, University of Queensland, Centre for Animal Science, St Lucia QLD 4072, Australia; B.Venus@uq.edu.au

**Keywords:** babesiosis, carrier, diagnosis, qPCR, cytochrome *b*, genotyping, ITS1, vaccines

## Abstract

*Babesia* spp. are tick-transmitted haemoparasites causing tick fever in cattle. In Australia, economic losses to the cattle industry from tick fever are estimated at AUD$26 Million per annum. If animals recover from these infections, they become immune carriers. Here we describe a novel multiplex TaqMan qPCR targeting cytochrome *b* genes for the identification of *Babesia* spp. The assay shows high sensitivity, specificity and reproducibility, and allows quantification of parasite DNA from *Babesia bovis* and *B. bigemina* compared to standard PCR assays. A previously published cytochrome *b* SYBR Green qPCR was also tested in this study, showing slightly higher sensitivity than the Taqman qPCRs but requires melting curve analysis post-PCR to confirm specificity. The SYBR Green assays were further evaluated using both diagnostic submissions and vaccinated cattle (at 7, 9, 11 and 14 days post-inoculation) showed that *B. bigemina* can be detected more frequently than *B. bovis*. Due to fewer circulating parasites, *B. bovis* detection in carrier animals requires higher DNA input. Preliminary data for a novel fluorescent PCR genotyping based on the Internal Transcribed Spacer 1 region to detect vaccine and field alleles of *B. bovis* are described. This assay is capable of detecting vaccine and novel field isolate alleles in a single sample.

## 1. Introduction

*Babesia* species are tick-borne intra-erythrocytic apicomplexan parasites found in a variety of domestic and wild animals, and in humans. Mixed infections are responsible for widespread morbidity and mortality in livestock of tropical and subtropical regions of the world [1]. Economic losses in cattle due to tick fever (babesiosis and anaplasmosis) are estimated at AUD$26 Million and >US$10 Billion in Australia [2] and worldwide [3] per annum, respectively. The standard method for diagnosis of clinical babesiosis is the microscopic examination of blood smears. Although this method is rapid, inexpensive, and reasonably sensitive in clinical cases, the sensitivity is poor for immune carrier animals because of the low parasitaemia in these animals [4,5]. Serological assays are useful for epidemiological studies and to confirm responses to vaccination, but do not indicate if the animal is persistently infected. Quantitative PCR may be useful for the detection of these latent carrier animals.

Live vaccines for cattle to protect against babesiosis and anaplasmosis have been produced in several countries including South Africa, Israel, Argentina and Australia [6,7]. Following vaccination, the parasite numbers decrease to carrier state levels and are usually no longer detectable in blood smears. In Australia, Tick Fever Centre (TFC) in addition to the production of live tick fever vaccines, also provides diagnostic support and investigations into outbreaks of tick fever in cattle, using microscopy and serology as the principal diagnostic tools. However, quantitative PCR (qPCR) is also a useful tool in this context to discriminate between *Babesia bovis* and *Babesia bigemina* infections at very low parasitaemias detected with light microscopy; and for genotype discrimination of the vaccine strain *B. bovis* from field isolates.

PCR assays are more sensitive than microscopic detection which cannot detect *Babesia* parasites at early stages of infection and also from immune carrier animals with low levels of parasitaemia [5,8,9]. Several conventional PCR assays have been employed in diagnostic applications and epidemiological studies to detect these parasites with a high degree of sensitivity [10,11,12], but these do not permit the estimation of the initial concentration of target DNA and are at best semi-quantitative [13]. Quantitative PCR has been used to assess disease severity and treatment outcome in various protozoan diseases [14,15]. SYBR Green qPCR detection of cytochrome *b* genes [16] and TaqMan-based qPCRs detecting 18S ribosomal genes [17] were reported for the detection and quantification of both *B. bovis* and *B. bigemina* from Spain and Argentina, and Brazil, respectively.

PCR methods have been invaluable for the differentiation of attenuated vaccine and field isolates supporting tick fever live vaccine production facilities in Australia [18,19], South Africa [20], and Israel [21]. The most significant *Babesia* spp. associated with these vaccine studies has been *B. bovis*. In the Australian context, *B. bovis* causes a much higher incidence of field outbreaks compared with *B. bigemina*; clinical disease associated with *B. bigemina* is diagnosed only occasionally. Previous typing studies have focused on single copy genes which may vary due to immune pressure such as the Bv80 and BvVA1 genes [13,20]. A tandem repeat-based multilocus typing system based on 14 *B. bovis* mini- and micro-satellites was developed demonstrating geographic strain differences, but vaccine and field isolate differentiation was not reported [22].

This paper describes the optimization and development of qPCRs for the identification and differentiation of *Babesia* spp. (*B. bovis* and *B. bigemina*) using Australian isolates. The new multiplex TaqMan qPCR assay was designed to amplify the mitochondrial DNA cytochrome *b* genes known to have a higher copy number compared with ribosomal RNA genes [23]. Preliminary data for a new genotyping method for *B. bovis* comparing vaccine and field isolates by amplifying the Internal Transcribed Spacer Sequence 1 region using a novel fluorescence PCR method are also reported [24] analyzed by capillary electrophoresis [25]. This novel data are compared to the single copy BvVA1 PCR assay which is one of the methods routinely used by TFC for the differentiation of vaccine and field isolates.

## 2. Materials and Methods

### 2.1. Parasite and Parasite DNA Samples

One isolate of both *B. bovis* Dixie strain and *B. bigemina* strain G previously identified by blood smears, standard PCR and sequencing techniques [13], were used as reference strains in the evaluation of all PCR methods described here. *Babesia bovis* and *B. bigemina* field strains were either extracted from diagnostic submissions or from previous diagnostic submissions which had been cryopreserved as stabilates at Tick Fever Centre (TFC) as previously described [19]. *Babesia bovis* diagnostic submissions were also presented to TFC as blood smears (venous blood or capillary blood from organs) on glass slides, or 10 mL blood vacutainers (BD Australia). Vacutainers were also collected from 17 calves vaccinated with Trivalent Tick Fever Vaccine (a live vaccine made with *B. bovis* Dixie strain, *B. bigemina* G strain and *Anaplasma marginale* subsp. *centrale*) at Days 7, 9, 11 and 14 post-inoculation to compare standard PCR with qPCRs in detecting *Babesia* parasites (68 samples).

### 2.2. DNA Isolation and Quantification from Purified Parasites

*Babesia bovis* (Dixie) and *B. bigemina* (G strain) positive control parasites were extracted from 75 mL of blood of known parasitaemia. *Babesia bigemina* and *B. bovis* infected blood samples were leucocyte filtered using Imugard III blood filters (Terumo BCT, Lakewood, CO, USA) according to the manufacturer’s instructions. Parasites were purified following the OIE method for *B. bovis* parasite antigen preparation [26]. Parasite DNA was extracted from purified *B. bovis* and *B. bigemina* parasites using the QIAamp DNA Blood Mini Kit (QIAGEN Pty Ltd., Victoria, Australia) according to the manufacturer’s instructions and then quantified by NanoDrop 2000 (Thermofisher Scientific Australia Pty Ltd., Victoria, Australia). DNA was subjected to 10-fold dilutions from 10 ng/µL to 1 ag/µL (*B. bovis*, approximately 3.5 × 10^6^ parasites/10 ng/µL; *B. bigemina*, approximately 2 × 10^6^ parasites/10 ng/µL). All dilutions of infected red blood cells were prepared in duplicate for the TaqMan qPCR assay in parallel to the SYBR Green qPCR assay and the standard PCR assays. Negative control DNA was isolated from the blood of uninfected cattle.

Purified DNA from *B. microti*, *B. duncani* and an unknown *Babesia* spp. were provided by the Centers for Disease Control and Prevention (Atlanta, GA, USA) to our laboratory previously and utilized as negative controls to evaluate the new PCR assays. DNA from *Anaplasma marginale* (Dawn strain) and *Anaplasma marginale* subsp. *centrale* (anaplasmosis vaccine strain [27]) was prepared from 10 mL EDTA vacutainers. Briefly, the blood samples were centrifuged at 2500× *g* for 10 min at room temperature, and the plasma and buffy coat layers removed by pipetting. Subsequently 200 µL of the red blood cell pellet was used to extract DNA using the QIAamp DNA Blood Mini Kit (QIAGEN Pty Ltd., Victoria, Australia) according to the manufacturer’s instructions.

### 2.3. DNA Isolation from Clinical Samples and Vaccinated Cattle

Preparation of samples containing parasites differed depending on the source of the sample which included fresh blood (10 mL EDTA or lithium heparin vacutainers), blood smears on glass slides (for *B. bovis* field isolates) and cryopreserved stabilates. For the vacutainers, the bloods were centrifuged to allow removal of the plasma and buffy coat, and processed through the QIAamp DNA Blood Mini Kits as described above for *Anaplasma* spp. For blood smears, the cells were scraped from glass slides using a scalpel blade and re-suspended in 200 µL of phosphate buffered saline (PBS) which was subsequently also processed using the QIAamp DNA Blood Mini Kits. For stabilate DNA extractions using cryopreserved blood samples (2.5 mL parasite infected blood mixed with 2.5 mL 20% polyvinyl-pyrrolidone-40 in PBS stored in liquid nitrogen), the 5 mL stabilate was first centrifuged at 4000× *g* for 10 min prior to removal of the supernatant, with the pellet re-suspended in 1 mL PBS and re-centrifuged at 10,000× *g* for 5 min prior to suspension in 200 µL PBS and extracted using the QIAamp DNA Blood Mini Kit (QIAGEN). For the evaluation of the *B. bovis* genotyping assay, DNA from seven characterized TFC *B. bovis* stabilates (Table 1) were extracted as described above. Stabilates each have an identifier within TFC’s database described as a capital letter followed by 2 digits e.g., C48, F95, and H03, as shown in Table 1. Table 1 (and Table S1) summarizes the sources and the DNA extraction methods used for all reference and field isolates to confirm assay specificities.

For the extraction of DNA from the 17 vaccinated cattle at 4 time points (*n* = 68), the same method for 10 mL EDTA vacutainers and the QIAGEN DNA Blood Mini Kits was undertaken, as described above. DNA concentrations were determined using the NanoDrop 2000 (ThermoFisher Scientific Australia Pty Ltd., Victoria, Australia). DNA from blood smears and stabilates were not measured due to the presence of bovine DNA (white blood cells) in the samples and thus would not be an accurate measure of parasite DNA.

### 2.4. Standard PCR

Standard PCRs for the detection of *B. bovis* and *B. bigemina* were performed using a Corbett Palm Cycler (QIAGEN Pty Ltd., Victoria, Australia). For *B. bovis*, a 25 μL reaction included 2.5 μL 10× *Taq* Buffer (5 PRIME GmbH, Hilden, Germany), 10 pmol of each primer (Bo Fwd and Bo Rev) [10], 200 μM dNTPs, 0.2 μL *Taq* DNA Polymerase (5 PRIME GmbH, Hilden, Germany) and 1–5 μL DNA template. Temperature cycling conditions were: 60 s at 95 °C, followed by 35 cycles of 60 s at 95 °C, 60 s at 55 °C and 90 s at 73 °C, with the final cycle with an additional 5 min at 73 °C.

For *B. bigemina* standard detection PCR, a 25 μL reaction included 2.5 μL 10× *Taq* Buffer (5 PRIME), 25 pmol of each primer (BiIA and BiIB) [28], 200 μM dNTPs, 0.5 μL *Taq* DNA Polymerase (5 PRIME) and 1–5 μL DNA template. Temperature cycling conditions were: 5 min at 95 °C prior to the addition of *Taq* polymerase, followed by 35 cycles of 60 s at 95 °C, 60 s at 65 °C and 90 s at 73 °C, with the final cycle with an additional 15 min at 73 °C.

Both *B. bigemina* and *B. bovis* standard PCR products were separated on 1% agarose and 1× TBE (45 mM Tris-borate and 1 mM EDTA pH 8) gels at 80 volts for 45 min, and visualized under UV following 5 μg/mL ethidium bromide staining. The molecular weight marker was the HyperLadder II (Bioline Australia Pty Ltd., Narellan, NSW, Australia).

### 2.5. Optimization of SYBR Green Based Quantitative PCR Assay

Primers used for the SYBR Green qPCR assays were as described by Buling and co-workers [16] to specifically amplify the mitochondrial DNA cytochrome *b* genes of *B. bovis* and *B. bigemina*. The pairs of forward and reverse primers are shown in Table 2. PCR cycling conditions were modified and optimized using the reagents and equipment commonly used at the TFC laboratory. Briefly, SYBR Green based qPCR was conducted in a Rotor-Gene 6000 (QIAGEN Pty Ltd., Victoria, Australia). Reactions (20 µL) included 10 µL of SensiMix 2× SYBR no-ROX kit (Bioline Australia Pty Ltd., Sydney, NSW, Australia), 500 nM of each oligonucleotide primer and 1 µL of extracted genomic DNA template. Temperature cycling conditions were: 5 min at 95 °C, followed by 40 cycles of 30 s at 95 °C, 30 s at 56 °C, and 30 s at 72 °C, acquiring SYBR Green fluorescence at the end of each extension step. After preliminary testing, the threshold was set to 0.2 for all assays. Cycle threshold (*C*t) scores, corresponding to the cycle number at which the amplification curve intersects the threshold line, were recorded for each sample. Negative (no template or DNA extraction kit buffer) controls were included in each PCR run. Melting curve analysis was performed using the manufacturer’s standard protocol (Bioline Australia Pty Ltd., Alexandria, NSW, Australia). A bovine mitochondrial 16S rRNA as a housekeeping gene was used to optimize the SYBR Green qPCR conditions [29].

### 2.6. TaqMan qPCR Assay

Species-specific primers (forward and reverse) and fluorescence-labelled probes for the multiplex qPCR assay were designed using Primer 3 software (http://primer3.sourceforge.net; Howard Hughes Medical Institute, Chevy Chase, MD, USA). The VIC and FAM labelled probes specifically target the mitochondrial cytochrome *b* genes of *B. bovis* and *B. bigemina*, respectively. All primers utilized in this study were synthesized through Integrated DNA Technologies (Skokie, IL, USA). TaqMan probes (VIC and FAM labelled) were synthesized through Applied Biosystems (ThermoFisher Scientific Pty Ltd., Victoria, Australia).

TaqMan qPCR was conducted in a Rotor Gene Q (QIAGEN Pty Ltd., Victoria, Australia). Reactions (20 µL) included 10 µL of SensiFAST 2× Probe Mix (Bioline Australia Pty Ltd., Alexandria, NSW, Australia), 500 nM of each oligonucleotide primer, 150 nM of the VIC- (*B. bovis*) or 6FAM- (*B. bigemina*) labelled probe, and 1 µL of extracted genomic DNA template. Temperature cycling conditions were: 5 min at 95 °C, followed by 40 cycles of 95 °C for 30 s, 54 °C for 30 s and 72 °C for 30 s, acquiring fluorescence on both the green (FAM) and yellow (VIC) channels at the end of each extension step. *C*t scores were recorded for each sample. Negative controls (no template or DNA extraction kit buffer) were included in each PCR run.

### 2.7. Sensitivity and Specificity of Species Specific Assays

Sensitivity of each assay was determined by using the serial 10 fold dilutions of DNA prepared from the control *B. bovis* and *B. bigemina* strains (Dixie and G strains, respectively) described above. To evaluate the analytical specificity, DNA from *Babesia microti*, *Babesia duncani*, *Anaplasma marginale*, and *Anaplasma marginale* subsp. *centrale* (vaccine strain for bovine anaplasmosis used in Australia, originally isolated in South Africa [30,31]) and DNA from non-infected bovine blood, were subjected to the qPCR assays for bovine *Babesia* parasites. The reference isolates are listed in Table 1.

### 2.8. Clinical Evaluation of Assay Sensitivity and Specificity of Species Specific Assays

Clinical sensitivity and specificity of the standard PCRs, TaqMan and SYBR Green qPCRs were evaluated using diagnostic samples submitted to TFC. Thirty-two *B. bovis* diagnostic samples and 14 *B. bigemina* stabilates (stored field samples) were screened (Table 1). In addition, the standard PCR and SYBR Green qPCR were used to screen 17 animals inoculated with both Australian vaccine strains (*B. bovis* Dixie strain and *B. bigemina* G strain) at 4 different time points (Days 7, 9, 11 and 14) post-inoculation (*n* = 68 samples). DNA prepared from the *B. bovis* Dixie and *B. bigemina* G strains described in 2.2 above were included as positive controls in these experiments.

### 2.9. Statistical Analysis

The Student’s *t*-test (tails 1, type 2) was used to compare the melting temperatures of the different amplification products from the species specific SYBR Green qPCR. Significance was set at *p* < 0.05.

### 2.10. Babesia bovis ITS Genotyping Method

New primers were designed flanking the ITS1 variable length region using a conserved forward primer and a *B. bovis* specific reverse primer to amplify fragments of ~200–250 bp depending on the isolate (Table 1). The forward primer, M13BbovITSF, is anchored in the 3’ end of the 18S rRNA gene. The forward primer was given an M13 extension so that an additional 6-FAM-labeled M13 forward primer could be included in the reaction for product detection [24]. The reverse primer, BbovITSR, was anchored in the conserved ITS1 3’ end flanking the variable regions of the ITS1. A Multiplex PCR kit (QIAGEN Pty Ltd., Victoria, Australia) was used to amplify 2 µL of DNA purified from previously cryopreserved stabilate samples as described above. PCRs were conducted in 12 µL volumes using 0.2 pmol M13Bbov ITSF, 2 pmol M13FAM, 2 pmol BbovITSR, 1.2 µL 5× Q solution and 6 µL QIAGEN Master Mix 2.5× (QIAGEN Pty Ltd., Victoria, Australia). The thermo-cycling conditions were as previously described: 95 °C 15 min, 94 °C 30 s, 50 °C 45 s, 72 °C 90 s for 35 cycles followed by 72 °C for 45 min in DNA Engine Peltier thermocycler (BioRad, Gladesville, NSW, Australia). The subsequent analyses of these PCR products were undertaken through the Animal Genetics Laboratory, the University of Queensland as an external service provider (http://animal-genetics.lab.uq.edu.au/). Briefly, the PCR products were diluted 1 in 10 with water and 3 µL of each diluted product mixed with 10 µL of formamide plus LIZ 500 size standard (1:0.004 ratio) (Applied Biosystems, Foster City, CA, USA). Samples were denatured at 95 °C for 3 min and then chilled on ice before being electrokinetically injected into a POP-7 polymer matrix using a 50 cm, 16 capillary, 3130XL DNA Genetic Analyzer (Applied Biosystems, Foster City, USA). Allele sizes were determined using Genemapper (v3.7, Applied Biosystems, Foster City, USA) as described previously [25].

## 3. Results

### 3.1. Sensitivity and Specificity

**Figure 1:**
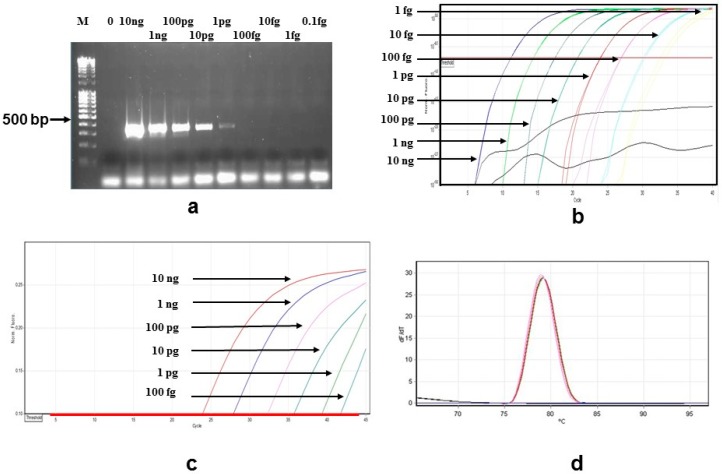
Results obtained from all *B. bovis* PCR assays examined in this study: (**a**) Sensitivity of the standard PCR assay determined following agarose gel electrophoresis. M is the Bioline HyperLadder II and the concentrations of *B. bovis* DNA are shown above each lane with PCR products estimated at 356 bp; (**b**) SYBR Green qPCR plots of *B. bovis* dilutions with DNA concentrations indicated; (**c**) TaqMan qPCR plots of *B. bovis* dilutions with DNA concentrations indicated; (**d**) Melting curves from the SYBR Green qPCRs in Figure 1b.

To evaluate the sensitivity of both SYBR Green and the TaqMan qPCRs (this study), serial dilutions of DNA extracted from *B. bovis* and *B. bigemina*-infected bovine blood were used. Sensitivity of *B. bovis* amplifications was 1 pg with standard PCR (Figure 1a), 1 fg with SYBR Green qPCR (Figure 1b) and 100 fg with TaqMan qPCR (Figure 1c). This corresponded to 35 *B. bovis* parasites/µL and 0.35 *B. bovis* parasites/µL for TaqMan and SYBR Green detection limits, respectively. Melting curve analysis for the SYBR Green qPCRs is shown in Figure 1d, at 79.06 ± 0.05 °C. No positive signal was observed in qPCR assays with DNA from other species listed in Table 1 (See Table S1).

**Figure 2:**
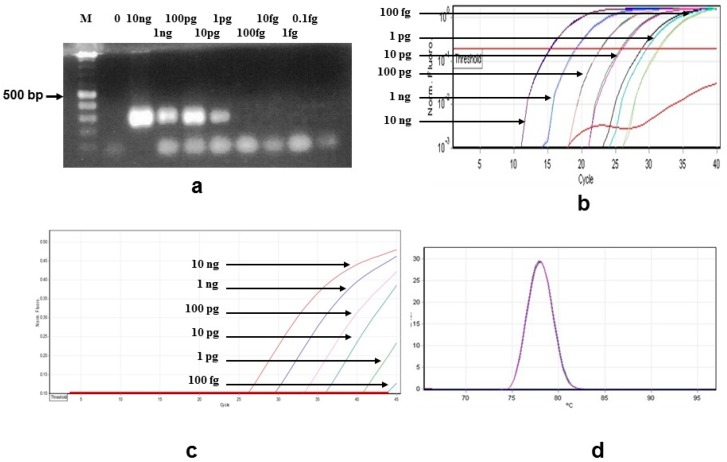
Results obtained from all *B. bigemina* PCR assays examined in this study: (**a**) Sensitivity of the standard PCR assay determined following agarose gel electrophoresis. M is the Bioline HyperLadder II and the concentrations of *B. bigemina* DNA are shown above each lane with PCR products estimated at 278 bp; (**b**) SYBR Green qPCR plots of *B. bigemina* dilutions with DNA concentrations indicated; (**c**) TaqMan qPCR plots of *B. bigemina* dilutions with DNA concentrations indicated; (**d**) Melting curves from the SYBR Green qPCRs in Figure 2b.

Sensitivity of *B. bigemina* amplifications was 10 pg with standard PCR (Figure 2a), 100 fg for both the SYBR Green (Figure 2b) and the TaqMan qPCRs (Figure 2c). For the qPCRs, this corresponds to the detection of 2 *B. bigemina* parasites/µL. Melting curve analysis for the SYBR Green qPCRs is shown in Figure 2d, at 77.83 ± 0.09 °C. Although *C*t values were observed with *B. microti* (5 isolates at Ct values higher than 35) and *B. duncani* (1 isolate, *C*t = 34.81) in the *B. bigemina* SYBR Green qPCR, the melting curve analysis showed that these other non-specific species have melting temperatures of 81.7 ± 0.95 °C (data not shown). TaqMan qPCR results were negative for all species listed in Table 1 including *B. microti* and *B. duncani* (See Table S1).

Melting curve analysis of the SYBR Green qPCR discriminated between *B. bigemina*- and *B. bovis*-infected samples (Figure 1d and Figure 2d). A student’s *t*-test found a significant difference between the melt temperatures of the two species. The melting temperature for *B. bigemina* was 77.83 ± 0.09 °C (*n* = 9), and for *B. bovis* was 79.06 ± 0.05 °C (*n* = 9, *p* < 0.001; Student’s *t*-test).

A summary of assay sensitivities is presented in Table 3 which compares published methods with that obtained here including our new Multiplex TaqMan qPCR targeting the cytochrome *b* genes of *B. bigemina* and *B. bovis*. The Buling et al. [16] SYBR Green qPCR was not as sensitive when applied to our samples in this study with 10-fold and 100-fold less detection for *B. bovis* and *B. bigemina* respectively. Buling et al. claim the detection of 1000 target copies using PCR products and not genomic DNA for this calculation. The multiplex TaqMan qPCR assay targeting the cytochrome *b* genes was also less sensitive compared to Kim et al. [17] targeting 18S rRNA genes however our parasite count would not be accurate as it was estimated from initial parasitaemias from large volumes of blood processed to isolate pure parasites. Overall specificities are comparable at 100%.

### 3.2. Clinical Sensitivity of SYBR Green qPCR Assays

Clinical sensitivity was determined by screening field samples from babesiosis diagnostic submissions as well as monitoring cattle vaccinated with *B. bovis* Dixie strain and *B. bigemina* G strain at four different time points post-inoculation. Table 4 summarizes the results showing that the SYBR Green qPCRs were able to detect each species from all clinical samples. The *B. bovis* standard PCR had negative results for 9/31 samples whereas the *B. bigemina* standard PCR assay amplified all 14 samples successfully. In addition, some of these diagnostic samples demonstrated mixed infections of *B. bovis* and *B. bigemina* as determined by qPCR. Supplementary Table S1 details results across reference and field isolates (31 *B. bovis* and 14 *B. bigemina*) for standard PCR, TaqMan and SYBR Green qPCRs. Results show mixed infections in four and six of the *B. bigemina* and *B. bovis* field samples, respectively. For the *B. bigemina* samples, two samples with a known history of tick fever vaccination (Table 1) were not positive in *B. bovis* qPCR (Table S1), indicating the lack of detection of carrier level parasites.

The standard PCR and SYBR qPCR assays were also used to detect *B. bovis* and *B. bigemina* in 17 vaccinated cattle sampled at four different time points post-inoculation (Days 7, 9, 11, 14) (see Table S2). All 17 animals were positive in *B. bigemina* qPCR at three or four time points. Only three animals were negative at Day 7, with the remaining 14 positive for qPCR at each time point. The *B. bigemina* standard PCR had 33 negative results out of the total 68 samples from the 17 cattle. For *B. bovis* SYBR Green qPCR, one animal was negative at all time points, two animals positive at one time point only, five positive at two time points, and nine with three or four positive time points per animal. In most cases, the negative time points were Day 7 and Day 9. The total number of positive *B. bovis* qPCR results was 25/68, which is much less than for *B. bigemina* with 65/68 positive. When the *B. bovis* standard PCR was correlated with the SYBR Green qPCR results, a further eight time points were negative where qPCR was positive, with only 17 positive samples overall by standard PCR. The results from this vaccination study indicate that the *B. bovis* qPCR is less sensitive “clinically” for the detection of *B. bovis* in comparison with *B. bigemina* detection.

### 3.3. Babesia Bovis ITS Genotyping

Allele sizes are summarized in Table 5. The current Australian *B. bovis* Dixie vaccine strain produces two alleles at 224 and 234 bp, which correspond to BvVA1 alleles at 3.5 and 6.8 kb, respectively. Two stabilates derived from this vaccine following tick transmission shows that one of these has a further two new alleles at 233 and 243 bp with extra alleles previously not detected using BvVA1 PCR (Table 5). *B. bovis* H97, J40, 32 and 34 are field isolates which show that these isolates also contain Dixie vaccine strain alleles detected using the ITS assay. Using the BvVA1 PCR, one *B. bovis* Dixie allele was also detected in strain H97. H92 is a field isolate from an animal with no vaccination history; hence the presence of one of the “Dixie through ticks” ITS1 allele is likely to be a coincidence. Note no BvVA1 Dixie alleles are detected in H92. Field isolate J50 appears to contain alleles commensurate with Dixie vaccine as well as an additional allele seen following tick transmission of Dixie vaccine (see H10). Isolate 33 did not yield positive BvVA1 results however using the ITS assay several alleles were detected with one matching a Dixie vaccine allele. *Babesia bigemina* and *A. marginale* did not produce PCR products in this assay (data not shown). Supplementary Figure S1 shows an example of GeneMapper plots for Dixie vaccine and H97 field isolate. Note that the molecular weights for Dixie vaccine were recorded at 225 and 235 bp for this particular analysis. Each analysis can vary by 1–2 bp, thus the incorporation of appropriate sample controls is recommended for each run to enable within run comparisons.

## 4. Discussion

We investigated the development of a novel multiplex TaqMan qPCR for the detection of *B. bovis* and *B. bigemina* simultaneously. The SYBR Green qPCR (also targeting the cytochrome *b* gene) appeared to be slightly more sensitive than our new multiplex assay. We also report preliminary data for a novel fluorescent PCR to genotype *B. bovis* targeting the ITS1, moreover detecting both vaccine and field alleles in single samples for the first time in Australia.

The detection of *Babesia* spp. in vaccinated cattle and disease outbreaks, and in immune carrier animals, can be problematic when low numbers of parasites are present. In comparison with conventional PCR assay, SYBR Green and Taqman qPCR assays have several advantages for detection and quantification of parasite DNA. Detection and quantification of a qPCR product can take place in a single tube, obviating the need for post-PCR manipulation and thus reducing the risk of contamination [33]. It is quick, taking less than three hours to set up, perform the assay and analyse the data; and primers for both *B. bovis* and *B. bigemina* can be added in one multiplex qPCR reaction. Therefore, qPCR has a faster turnaround time than the conventional PCR assay.

Our study confirmed that qPCRs are more sensitive than standard PCRs, being able to detect *Babesia* infection in bovine blood at 1000-fold lower concentrations than standard PCR. The cytochrome *b* gene was targeted due its reported higher copy number than ribosomal genes [23]. The same detection levels reported by Buling et al. [16] were not achieved for the SYBR Green qPCR, particularly for *B. bigemina*. Some of these differences could be attributed to the use of different reagents, thermocyclers, DNA extraction protocols and the samples used to calculate assay sensitivities. Specifically, Buling et al. [16] utilized DNA from species specific PCR products to calculate DNA concentrations for their assay sensitivity calculations. In comparison, we determined the DNA concentration using genomic parasite DNA extracts which would explain the decrease in sensitivity for our samples. A 10-fold decrease in the number of *Babesia* parasites was detected in our TaqMan assays compared with the published protocol [17]. Kim et al. [17] processed small volumes of parasites from in vitro culture using minimal steps compared to our extraction procedure and targeted 18S rRNA genes. Our parasite numbers would have been over-inflated as we first purified parasites from large volumes of infected bovine blood (~400 mL) taken directly from our vaccine donor animals. Subsequently a subset of these purified parasites (equating to ~75 mL of infected blood) were used to purify DNA and the number of parasites screened in the cytochrome *b* TaqMan PCRs was “extrapolated”. It is known that the purification of parasites from whole blood leads to loss of parasites through the centrifugation steps thus our extrapolated parasite count is far from accurate. Overall, the SYBR Green assays appeared to be more sensitive than the TaqMan assays for the same target, melting temperature analysis is needed for SYBR Green specificity which could be problematic for some diagnostic laboratories. It thus may be more beneficial to use the multiplex TaqMan assay (described here) with higher template DNA in order to obtain clearer results and better robust diagnostic specificity. Standardization of assays between laboratories would be best achieved by sharing samples.

This study also demonstrated the successful detection of *B. bovis* using DNA preparations from blood smears. This was undertaken using Giemsa’s stained smears where microscopy could not differentiate *Babesia* species; or the presence of *Babesia* species was suspected, but was not certain, due to smear quality. qPCR detection of *B. bovis* parasites was successful from smear material which provides another option for diagnosticians when blood smears cannot be interpreted. This is the first time that DNA and PCR technology has been applied for the diagnosis of bovine babesiosis using stained blood smears, to our knowledge.

Recently, a nested PCR targeting the cytochrome *b* genes from both *B. bovis* and *B. bigemina* detected as low as 0.1 fg DNA [34]. Nested PCRs thus can have similar sensitivity to real time or qPCRs and have been applied in the investigation of bovine babesiosis pathogens in the Philippines, Syria, Vietnam, Thailand and Kenya [35,36,37,38]. However, nested PCR assays are at high risk of introducing PCR product contamination, hence our evaluation using TaqMan and SYBR Green qPCR methods.

This study also showed that in clinical samples, the detection of *B. bovis* can be compromised due to lower parasitaemia compared with *B. bigemina*. It is known that *B. bovis* parasitaemia during acute infection can be lower than 1% compared with *B. bigemina* at >10% [26]. We thus recommend for qPCR of *B. bovis*, that a higher concentration of DNA be used as template. As we routinely purified 200 µL from a RBC pellet prepared from 10 mL blood tubes, we recommend that for *B. bovis* qPCR that the complete RBC pellet be processed and concentrated prior to addition as PCR template. This would increase the ability to detect *B. bovis* in immune carriers, as demonstrated in the vaccine qPCR study and in field isolates known to have been vaccinated where *B. bovis* could not be detected using the methods described.

In this study we employed a novel fluorescence technology for the discrimination of PCR fragments ranging from 142–337 bp using capillary electrophoresis. We verified the presence of allele variants for three of the *B. bovis* isolates (Dixie vaccine, H97 and J40) through sequencing (data not shown). This method was originally described by Schuelke (2000) reporting the usefulness of this approach as an alternative to agarose gel electrophoresis which usually cannot discriminate similarly sized fragments [24]. Godwin and Morgan (2014) further demonstrated the ability of this technology to recognize 10 species of poultry *Eimeria* parasites targeting a non-coding mitochondrial genome region between the cytochrome c oxidase subunit 3 (Cox3) and the Large Subunit 1 ribosomal RNA genes [25]. Previous methods using PCR to target single copy genes were useful as they detected different populations of *B. bovis* [13]. The *B. bovis* internal transcribed spacer gene 1 (between the 18S and the 5.8S rRNA coding regions) has not been exploited for genotyping to our knowledge, however, as more than one rDNA copy is present per *Babesia* genome, it is feasible that the sensitivity will increase over single copy genes previously used (BvVA1, Bv80) [13].

This is thus the first time that *B. bovis* vaccine genotypes have been demonstrated in field isolates confirming the presence of the Australian *B. bovis* Dixie vaccine strain. This has been previously shown with the South African *B. bovis* vaccine using Bv80 gene genotyping and a cartridge gel system capable of separating fragments differing by 6–10 bp [39]. We also demonstrated the presence of two extra alleles in one sample of the *B. bovis* Dixie vaccine strain which had been passaged through ticks. The other “Dixie through ticks” sample did not demonstrate non-Dixie vaccine alleles which may be due to the concentration of DNA used in the assay. The concentration of DNA in all *B. bovis* PCRs appears to be a contentious issue and must be addressed by using higher template DNA concentrations in all *B. bovis* PCR assays. In our previous studies using Bv80 and BvVA1 genotyping PCRs, vaccine genotypes were not detected in vaccinated field samples possible due to the low numbers of parasites at these carrier levels [18]. The new fluorescent ITS PCR using capillary electrophoresis was able to confirm the presence of different alleles including vaccine genotypes, and thus is more sensitive than the previous gel electrophoresis methods reported [18]. Further research is needed to conclude whether the Australian vaccine strain is co-transmitted with field isolates by ticks as described recently for the South African live *B. bovis* vaccine [39].

## Figures and Tables

**Table 1:** Sources of reference isolates, *Babesia bigemina* and *Babesia bovis* vaccine and field samples used to determine specificity of the assays described in this study (details in Table S1).

Species Strain/No.	Sample Source ^1^
**Reference Isolates:**	
*B. microti*-1610	CDC
*B. microti*-1737	CDC
*B. microti*-1750	CDC
*B. microti*-1743	CDC
*B. microti*-1716	CDC
*B. duncani*-1671	CDC
*Babesia* spp. 1749 CDC	CDC
*A. marginale* subsp. *central* **^2^**	TFC vaccine
*A. marginale* Dawn strain	TFC stabilate
*B. bovis*-Dixie vaccine strain	TFC vaccine calf
*B. bigemina*-G vaccine strain	TFC vaccine calf
***B. bovis*** **and *B. bigemina* field isolates (Species specific qPCR assays):**	
*B. bovis*—1–31	TFC field samples
*B. bigemina*—1–14	TFC stabilates of field strains
***B. bovis*** **isolates for genotyping evaluation ^3^:**	
*B. bovis* Dixie vaccine passaged through ticks-H03 **^4^**, H10 **^4^**	TFC stabilates
*B. bovis* H92 **^5^**	TFC stabilates of field strains
*B. bovis* H97 **^6^**	
*B. bovis* J40 **^6^**	
*B. bovis* J50 **^6^**	
*B. bovis*—32 **^6^**	TFC field samples
*B. bovis*—33 **^5^**	
*B. bovis*—34 **^6^**	

**^1^** All samples were from Tick Fever Centre (TFC) except for these control species from the CDC: Centers for Disease Control and Prevention, USA; DNA provided from CDC to our laboratory; **^2^** Originally from the Onderstepoort Veterinary Institute, South Africa; collected from TFC calf animal number 9710; **^3^** Compared with positive control *B. bovis* Dixie vaccine strain; **^4^** *B. bovis* Dixie vaccine strain passaged through ticks; **^5^** Field isolate with no known *Babesia* spp. vaccination history; **^6^** Field isolate with known *Babesia* spp. vaccination history.

**Table 2:** Primers and probes for the PCR assays for *B. bovis* and *B. bigemina* used in this study.

Species	Primer/Probe	Sequence 5′-3′	Product Length (bp)	Target Sequence (Genbank No.)	Reference
*B. bovis*		Standard PCR	356	(M38218.1)	[10]
	BoF	CACGAGGAAGGAACTACCGATGTTGA		656–681	
	BoR	CCAAGGAGCTTCAACGTACGAGGTCA		986–1011	
		SYBR Green qPCR	88	(GQ214235.1)	[16]
	cbosg-1 (F)	TGTTCCTGGAAGCGTTGATTC		135–155	
	cbosg-2 (R)	AGCGTGAAAATAACGCATTGC		202–222	
		TaqMan qPCR	90	(AB499088.1)	This study
	bovisF160	ATATGTTTGCATTTGCTG		160–178	
	bovisR249	CTCCAAACCAATATGAAAG		230–249	
	bovisPb	VIC-CAAACCATAAAGTCATCGGTATATCCTAC-MGB		196–225	
		Genotyping PCR	245		This study (*B. bovis*) M13 [24]
	M13BbovITSF	**^1^** GAGCGGATAACAATTTCACACAGGAAGGAGAAGTCGTAACAAGG		(EF458299.1)	
	BbovITSR	GGTCGTGGCAGTCACGGC		9–28	
	M13-FAM	6FAM-GAGCGGATAACAATTTCACACAGG		236–253	
*B. bigemina*		Standard PCR	278	(S45366.1)	[28]
	BiIA	CATCTAATTTCTCTCCATACCCCTCC		6–31	
	BiIB	CCTCGGCTTCAACTCTGATGCCAAAG		258–283	
		SYBR Green qPCR	88	(GQ214234.1)	[16]
	cbisg-1 (F)	TGTTCCAGGAGATGTTGATTC		182–202	
	cbisg-2 (R)	AGCATGGAAATAACGAAGTGC		249–269	
		TaqMan qPCR	146	(AB499085.1)	This study
	bigemF295	GGTCTATTTGGTGGAGTT		295–313	
	bigemR413	ACAAGACCAAATGCAATT		395-413	
	bigemPb	6FAM-CAATTGTTCTTGGAGCAGCT-TAMRA		329–349	
Bovine		SYBR Green qPCR **^2^**	108	(V00654)	[29]
	MTFB	GCGATTTTAAAGACTAGACCC		2642–2662	
	MTRBO	TGAATAGGATTGCGCTGT		2732–2749	

**^1^** M13 sequence adaptor sequence underlined [24]; **^2^** SYBR green based on the bovine mitochondrial 16S rRNA gene used to optimize our SYBR Green assay conditions [29].

**Table 3:** Summary of sensitivity and specificity of published qPCR assays for the detection of *Babesia bovis* and *Babesia bigemina*.

Reference	Assay Type	Species	Target Gene	Specificity	Sensitivity	Source of Isolates
[16]	SYBR Green	*B. bovis* *B. bigemina*	cytochrome *b*	100%	0.1 fg **^1^** (1000 target copies)	Spain, Argentina
		*B. bovis*		100%	1 fg or 0.35 parasites/µL	Australia **^2^**
		*B.* *bigemina*		100%	100 fg or 20 parasites/µL	
This study	TaqMan probes	*B. bovis*	cytochrome *b*	100%	100 fg or 35 parasites/µL	Australia
		*B. bigemina*		100%	100 fg or 20 parasites/µL	
[32]	SYBR Green	*B. bovis*	*msa2c*	100%	1000 copies/mL	Brazil
[17]	TaqMan probes	*B. bovis* *B. bigemina*	18S rRNA genes	100%	2.5 parasites/µL	Brazil

**^1^** Parasite numbers per µL sensitivity was not determined; **^2^** Buling et al. method [16] using Australian samples from this study.

**Table 4:** Summary of clinical sensitivities for *Babesia bovis* and *Babesia bigemina* standard PCRs and SYBR Green qPCRs.

Clinical Samples ^1^	Standard PCR *B. bovis*	Standard PCR *B. bigemina*	SYBR qPCR *B. bovis*	SYBR qPCR *B. bigemina*
31 *B. bovis*	22/31	ND	31/31	6/31
14 *B. bigemina*	ND	14/14	4/14	14/14

**^1^** See Table 1 and Table S1 for further descriptions of the samples used.

**Table 5:** Summary of *Babesia bovis* genotyping showing BvVAI PCR results compared with ITS alleles determined following capillary electrophoresis and GeneMapper analysis.

*B. bovis* Isolates ^1^	Isolate Description	BvVA1 ^1,2^ kb	ITS Alleles ^1,2^ bp
Dixie	Vaccine isolate	**3.5**, **6.8**	**224**, **234**
H03	Dixie vaccine following tick passage	**6.8**	**224**, **234**
H10	Dixie vaccine following tick passage	**3.5, 6.8**	**224**, 233, **234**, 243
H92 **^3^**	Field isolate **^3^**	5.5, 6.1	218, 233, 235, 239, 242, 247
H97	Field isolate	6.4, **6.8**	**224**, **234**, 242
J40	Field isolate	6.1, (6.2)	**224**, 226, 227, 229, 231, **234**, 236, 238, 239, 242
J50	Field isolate	6.1	**224**, 233, **234**
32	Field isolate	6.2, 8.0	**224**, **234**, 237, (309), (337)
33	Field isolate	Nil	(142), (189), 220, **224**, 250, 256, 315, 325, 337, 342
34	Field isolate	6.5	218, (**224**), **234**, 244, (302), (337)

**^1^** Dixie vaccine strain alleles are shown in bold font; **^2^** Faint bands are indicated by a bracket; **^3^** No history of Dixie vaccine strain vaccination.

## Supplementary Materials

Supplementary materials can be found at www.mdpi.com/2306-7381/3/3/23/s1. Figure S1: Example Genemapper plots showing peaks for Dixie vaccine and one field isolate H97. Note fragment sizes 225 and 235 bp for Dixie vaccine strain for this electrophoresis run, Table S1: Comparison of standard PCR, TaqMan PCR and SYBR Green qPCRs for the detection of *B. bovis* and *B. bigemina* in reference isolates and field samples, Table S2: Use of standard PCR and SYBR Green qPCR to detect both *B. bovis* and *B. bigemina* in 17 vaccinated cattle at 4 time points post-inoculation (Days 7, 9, 11 and 14), total 68 samples.

## Abbreviations

The following abbreviations are used in this manuscript:

MDPI	Multidisciplinary Digital Publishing Institute
DOAJ	Directory of open access journals
TFC	Tick Fever Centre
PCR	Polymerase Chain Reaction
qPCR	Quantitative PCR
M13	M13 bacteriophage
EDTA	Ethylenediaminetetraacetic acid
PBS	Phosphate buffered saline
dNTPs	Deoxynucleotides
