# Supplementary material for: An Evaluation of Quantitative PCR Assays (TaqMan® and SYBR Green) for the Detection of Babesia bigemina and Babesia bovis, and a Novel Fluorescent-ITS1-PCR Capillary Electrophoresis Method for Genotyping B. bovis Isolates
Source: Vet Sci. 2016 Sep 13;3(3):23. doi: 10.3390/vetsci3030023 (PMC5606575; doi:10.3390/vetsci3030023)
Supplement: Supplementary file 1 [file vetsci-03-00023-s001.pdf]

Supplementary Materials: An Evaluation of Quantitative PCR Assays (TaqMan® and SYBR Green) for the Detection of *Babesia bigemina* and *Babesia bovis*, and a Novel Fluorescent-ITS1-PCR Capillary Electrophoresis Method for Genotyping *B. bovis* Isolates

Bing Zhang, Jacqueline L. Sambono, Jess A. T. Morgan, Bronwyn Venus, Peter Rolls and Ala E. Lew-Tabor

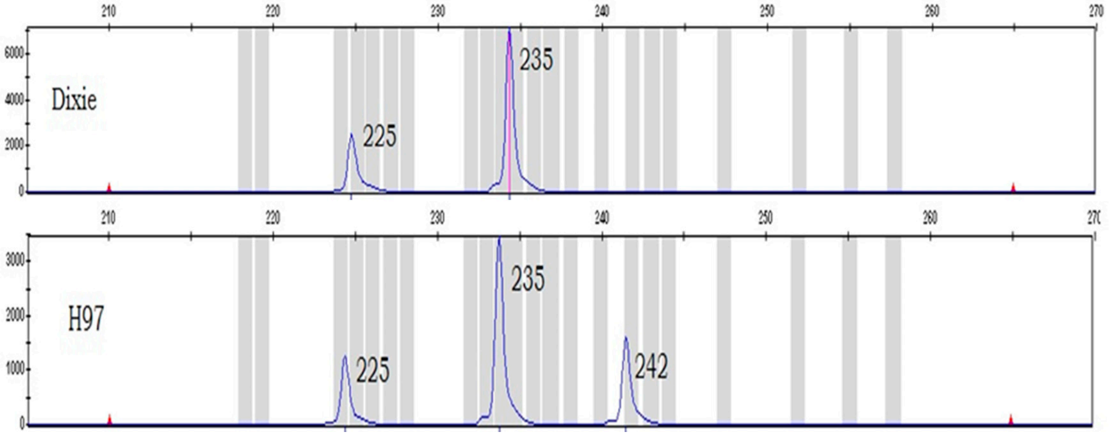

Figure S1. Example Genemapper plots showing peaks for Dixie vaccine and one field isolate H97. Note fragment sizes 225 and 235 bp for Dixie vaccine strain for this electrophoresis run.

Table S1. Comparison of standard PCR, TaqMan PCR and SYBR Green qPCRs for the detection of *B. bovis* and *B. bigemina* in reference isolates and field samples.

Isolate/Field Strains	Sample for Extraction	*Babesia bovis* PCRs			*Babesia bigemina* PCRs		
		Standard PCR	TaqMan PCR	SYBR PCR	Standard PCR	TaqMan PCR	SYBR PCR
B. microti-1610	DNA (CDC)	ND	negative	negative	ND	negative	negative
B. microti-1737	DNA (CDC)	ND	negative	negative	ND	negative	negative
B. microti-1750	DNA (CDC)	ND	negative	negative	ND	negative	negative
B. microti-1743	DNA (CDC)	ND	negative	negative	ND	negative	negative
B. microti-1716	DNA (CDC)	ND	negative	negative	ND	negative	negative
B. duncani-1671	DNA (CDC)	ND	negative	negative	ND	negative	negative
Babesia spp. 1749 CDC	DNA (CDC)	ND	negative	negative	ND	negative	negative
A. marginale var. *centrale*	200 µL RBC pellet from 10 mL blood tube	ND	negative	negative	ND	negative	negative
A. marginale Dawn strain	200 µL RBC pellet from 10mL blood tube	ND	negative	negative	ND	negative	negative
B. bigemina—G strain vaccine strain	Purified parasites from 75 mL blood	negative	negative	negative	positive	positive	positive
B. bovis—Dixie vaccine strain	Purified parasites from 75 mL blood	positive	positive	positive	negative	negative	negative
B. bovis—1	blood smear (venous)	positive	ND	positive	ND	ND	negative
B. bovis—2	blood smear (liver)	positive	ND	positive	ND	ND	positive
B. bovis—3	blood smear (spleen)	positive	ND	positive	ND	ND	negative
B. bovis—4	blood smear (kidney)	positive	ND	positive	ND	ND	negative
B. bovis—5	blood smear (ear)	negative	ND	positive	ND	ND	negative
B. bovis—6	blood smear (venous)	negative	ND	positive	ND	ND	negative
B. bovis—7		negative	ND	positive	ND	ND	negative
B. bovis—8		negative	ND	positive	ND	ND	negative
B. bovis—9		negative	ND	positive	ND	ND	ND
B. bovis—10		negative	ND	positive	ND	ND	positive
B. bovis—11		negative	ND	positive	ND	ND	negative
B. bovis—12	200 µL RBC pellet from 10 mL blood tube	positive	ND	positive	ND	ND	positive

Table S1. *Cont.*

Isolate/Field Strains	Sample for Extraction	*Babesia bovis* PCRs			*Babesia bigemina* PCRs		
		Standard PCR	TaqMan PCR	SYBR PCR	Standard PCR	TaqMan PCR	SYBR PCR
B. bovis—13	200 µL RBC pellet from 10 mL blood tube	positive	ND	positive	ND	ND	Negative
B. bovis—14		positive	ND	positive	ND	ND	negative
B. bovis—15		positive	ND	positive	ND	ND	negative
B. bovis—16		positive	ND	positive	ND	ND	positive
B. bovis—17		negative	ND	positive	ND	ND	negative
B. bovis—18		positive	ND	positive	ND	ND	negative
B. bovis—19		positive	ND	positive	ND	ND	negative
B. bovis—20		positive	ND	positive	ND	ND	negative
B. bovis—21		positive	ND	positive	ND	ND	negative
B. bovis—22		negative	ND	positive	ND	ND	negative
B. bovis—23		negative	ND	positive	ND	ND	negative
B. bovis—24		negative	ND	positive	ND	ND	negative
B. bovis—25		negative	ND	positive	ND	ND	negative
B. bovis—26		negative	ND	positive	ND	ND	negative
B. bovis—27		positive	ND	positive	ND	ND	negative
B. bovis—28		positive	ND	positive	ND	ND	negative
B. bovis—29		positive	ND	positive	ND	ND	positive
B. bovis—30	blood smear (venous)	positive	ND	positive	ND	ND	negative
B. bovis—31	200 µL RBC pellet from 10 mL blood tube	negative	ND	positive	ND	ND	positive
B. bigemina—G strain vaccine strain	Blood 75 mL	negative	negative	negative	positive	positive	positive
B. bigemina—1 (C48) [1]	whole blood stabilates 2.5 mL blood cryopreserved	ND	ND	positive	positive	positive	positive
B. bigemina—2 (F56) [1]		ND	ND	negative	positive	positive	positive
B. bigemina—3 (F95) [1]		ND	ND	negative	positive	positive	positive
B. bigemina—4 (G24) [1]		ND	ND	negative	positive	positive	positive
B. bigemina—5 (G30) [1]		ND	ND	negative	positive	positive	positive
B. bigemina—6 (G41) [2]		ND	ND	negative	positive	positive	positive

Table S1. *Cont.*

Isolate/Field Strains	Sample for Extraction	*Babesia bovis* PCRs			*Babesia bigemina* PCRs		
		Standard PCR	TaqMan PCR	SYBR PCR	Standard PCR	TaqMan PCR	SYBR PCR
B. bigemina—7 (G59) [2]	whole blood stabilates 2.5 mL blood cryopreserved	ND	ND	negative	faint positive	positive	Positive
B. bigemina—8 (H13) [1]		ND	ND	negative	positive	positive	positive
B. bigemina—9 (H17) [1]		ND	ND	negative	positive	positive	positive
B. bigemina—10 (H23) [1]		ND	ND	negative	positive	positive	positive
B. bigemina—11 (H46) [1]		ND	ND	positive	faint positive	positive	positive
B. bigemina—12 (H48) [1]		ND	ND	positive	positive	positive	positive
B. bigemina—13 (H51) [2]		ND	ND	positive	positive	positive	positive
B. bigemina—14 (H73) [2]		ND	ND	negative	positive	positive	positive

Orange highlight: Indicates mixed infection of *B. bovis* and *B. bigemina* detected using Qpcr; [1] field isolate, no Babesia vaccination history known; [2] field isolate with known *Babesia* spp. vaccination history.

Table S2. Use of standard PCR and SYBR Green qPCR to detect both *B. bovis* and *B. bigemina* in 17 vaccinated cattle at 4 time points post-inoculation (Days 7, 9, 11 and 14), total 68 samples.

Day	Animal No.	PCR Assay			
		Babesia bigemina		*Babesia bovis*	
		SYBR qPCR	std PCR	SYBR qPCR	std PCR
Day 7	9597	negative	negative	negative	negative
Day 9		positive	negative	negative	negative
Day 11		positive	positive	negative	negative
Day 14		positive	negative	positive	positive
Day 7	9598	positive	negative	negative	negative
Day 9		positive	negative	negative	negative
Day 11		positive	positive	positive	positive
Day 14		positive	positive	positive	positive
Day 7	9599	positive	positive	negative	negative
Day 9		positive	positive	negative	negative
Day 11		positive	negative	positive	positive

Table S2. *Cont.*

Day	Animal No.	PCR Assay			
		Babesia bigemina		*Babesia bovis*	
		SYBR qPCR	std PCR	SYBR qPCR	std PCR
Day 14		positive	negative	positive	Positive
Day 7	9600	positive	negative	positive	negative
Day 9		positive	positive	positive	positive
Day 11		positive	negative	positive	positive
Day 14		positive	negative	positive	positive
Day 7	9601	positive	positive	negative	negative
Day 9		positive	positive	positive	negative
Day 11		positive	negative	positive	positive
Day 14		positive	negative	positive	positive
Day 7	9602	positive	negative	positive	negative
Day 9		positive	positive	positive	negative
Day 11		positive	positive	positive	positive
Day 14		positive	negative	positive	positive
Day 7	9603	positive	positive	negative	negative
Day 9		positive	positive	positive	positive
Day 11		positive	negative	positive	positive
Day 14		positive	negative	positive	positive
Day 7	9604	positive	negative	negative	negative
Day 9		positive	negative	negative	negative
Day 11		positive	positive	negative	negative
Day 14		positive	positive	negative	negative
Day 7	9605	positive	negative	negative	negative
Day 9		positive	positive	negative	negative
Day 11		positive	positive	positive	negative
Day 14		positive	negative	positive	positive
Day 7	9607	positive	negative	positive	negative

Table S2. *Cont.*

Day	Animal No.	PCR Assay			
		Babesia bigemina		*Babesia bovis*	
		SYBR qPCR	std PCR	SYBR qPCR	std PCR
Day 9		positive	positive	positive	Positive
Day 11		positive	positive	positive	positive
Day 14		positive	negative	positive	positive
Day 7	9609	negative	negative	negative	negative
Day 9		positive	negative	negative	negative
Day 11		positive	positive	positive	negative
Day 14		positive	positive	positive	positive
Day 7	9610	positive	negative	negative	negative
Day 9		positive	positive	negative	negative
Day 11		positive	positive	positive	positive
Day 14		positive	negative	positive	positive
Day 7	9611	negative	negative	negative	negative
Day 9		positive	negative	positive	positive
Day 11		positive	negative	positive	positive
Day 14		positive	negative	positive	positive
Day 7	9614	positive	negative	negative	negative
Day 9		positive	positive	negative	negative
Day 11		positive	positive	positive	negative
Day 14		positive	faint positive	positive	positive
Day 7	9616	positive	positive	positive	positive
Day 9		positive	positive	positive	positive
Day 11		positive	positive	positive	positive
Day 14		positive	negative	positive	positive
Day 7	9618	positive	negative	positive	negative
Day 9		positive	negative	negative	negative
Day 11		positive	positive	negative	negative
Day 14		positive	positive	negative	negative

Yellow highlight indicates negative qPCR/standard PCR; orange highlight indicates negative standard PCR yet qPCR positive.
